# Sensitization patterns to cat molecular allergens in subjects with allergic sensitization to cat dander

**DOI:** 10.1002/clt2.12294

**Published:** 2023-08-12

**Authors:** Saliha Selin Özuygur Ermis, Aram Norouzi, Magnus P. Borres, Rani Basna, Linda Ekerljung, Carina Malmhäll, Emma Goksör, Göran Wennergren, Madeleine Rådinger, Jan Lötvall, Hannu Kankaanranta, Bright I. Nwaru

**Affiliations:** ^1^ Krefting Research Centre Institute of Medicine Sahlgrenska Academy University of Gothenburg Gothenburg Sweden; ^2^ Norvia Kliniken Solna Sweden; ^3^ ImmunoDiagnostics Thermo Fisher Scientific Uppsala Sweden; ^4^ Department of Maternal and Child Health Uppsala University Uppsala Sweden; ^5^ Department of Pediatrics Queen Silvia Children's Hospital University of Gothenburg Gothenburg Sweden; ^6^ Faculty of Medicine and Life Sciences University of Tampere Tampere Finland; ^7^ Department of Respiratory Medicine Seinäjoki Central Hospital Seinäjoki Finland; ^8^ Wallenberg Centre for Molecular and Translational Medicine Institute of Medicine University of Gothenburg Gothenburg Sweden

**Keywords:** allergic sensitization, cat allergy, Fel d 1, lipocalins, molecular allergens

## Abstract

**Background:**

The use of molecular allergology has increasingly become common in the diagnosis and management of allergic diseases. However, there is still a lack of data on cat molecular allergens in adults. Therefore, we aimed to uncover the sensitization patterns to cat molecular allergens.

**Methods:**

Participants were recruited from the West Asthma Sweden Study, a population‐based study enriched with asthma subjects aged 16–75 years. Of 1872, 361 individuals were positive for cat dander immunoglobulin E and were further analysed for cat molecular allergens (Fel d 1/2/4/7). Sensitization patterns were classified as monosensitization, polysensitization, and concomitant sensitization, and were related to demographic and clinical measurements.

**Results:**

Among cat‐sensitized subjects, 84.2% were sensitized to secretoglobin, while 42.4% were sensitized to lipocalins. Nearly half of the subjects were monosensitized to Fel d 1. Polysensitization was observed in 20.2%, and concomitant sensitization to protein families was seen in 7.2%. Asthma prevalence, cat exposure, and rural living were associated with poly‐ and concomitant sensitization to protein families. Concomitant sensitization to single allergens was more common in those with asthma than in those without, while concomitant sensitization to both Fel d 1 and Fel d 4 was the most common pattern in individuals with asthma. Sensitization patterns also differed according to cat ownership and the degree of urbanization.

**Conclusion:**

Sensitization to molecular allergens was observed in 90.9% of cat‐sensitized subjects and showed variations across participants' background characteristics and the presence of asthma. Identification of sensitization patterns to cat allergens might provide better characterization of cat‐allergic subjects.

## 1 INTRODUCTION

As cat ownership has become very widespread worldwide, domestic cats (*Felis domesticus*) are now one of the most common sources of indoor airborne allergens.^1^ Nearly a quarter of households in the United States owns a cat, with an average of 1.8 cats per household, while one in five households owns at least one cat in Sweden according to current data.^2^, ^3^ Additionally, subjects without a cat could also be exposed to cat allergens via secondhand transmission, such as in public transportation and schools.^4^, ^5^ Therefore, the development of cat allergy is also associated with the number of cat owners in the community besides the primary exposure at households.^6^, ^7^

The prevalence of cat sensitization in the United States is around 12% among subjects ≥6 years old.^8^ Cat allergy has also been increasing gradually and sensitization to cat dander increased from 16% to 26% between 1994 and 2009 in Sweden.^9^, ^10^, ^11^, ^12^, ^13^ Besides, cat allergy poses a high risk of the development of allergic diseases such as asthma and rhinitis.^1^, ^14^ With increasing prevalence and disease burden, there has been a growing interest in new diagnostic techniques to improve the diagnosis of cat allergy in recent years.^15^

Component‐resolved diagnostics (CRD) is an in vitro diagnostic method to determine the molecular basis of allergic sensitization.^16^, ^17^, ^18^ In CRD, specific immunoglobulin E (sIgE) levels against each allergen molecule, within an allergenic source, could be measured individually.^16^, ^18^, ^19^ To date, eight different cat molecular allergens have been registered with the World Health Organization/International Union of Immunological Societies (WHO/IUIS) Allergen Nomenclature database (Fel d 1 to Fel d 8) and four of them (Fel d 1, Fel d 2, Fel d 4, and Fel d 7) are available for clinicians.^20^, ^21^

Fel d 1 is a species‐specific cat allergen and has been shown to be a good marker for the cat allergy.^9^, ^22^ On the other hand, identification of other cat molecular allergens (Fel d 2, Fel d 4, and Fel d 7) could provide more detailed information about sensitization patterns and help to shed light on patient‐tailored risk profiles.^23^ As the number of people affected by cat allergy in the population is increasing,^13^ it is crucial to define patients' sensitization profiles, particularly at the molecular level, and delineate their clinical implications on allergic and respiratory diseases. This could both help to improve diagnostic algorithms as well as disease management strategies. Asarnoj et al suggested that monosensitization and polysensitization demonstrate distinct allergy phenotypes in cat and dog allergies, and polysensitization could predict the development of allergic diseases over time in children.^24^ Hence, physicians can make risk assessment according to sensitization profiles. Concomitant sensitization to Fel d 1 and Fel d 4 was previously associated with an increased risk of asthma in schoolchildren.^25^ Besides, sensitization to Fel d 2 or Fel d 4 was linked to respiratory symptoms and the severity of asthma in paediatric populations.^26^, ^27^, ^28^ However, these findings were mainly confined to schoolchildren or studies with small sample sizes; yet, limited data have been available for adults.

Although evidence about molecular allergens has been gradually expanding, there is still a lack of data about sensitization to the cat molecular allergens in adults. The aim of this study was to identify the sensitization profiles to cat molecular allergens of adults with cat allergy based on sensitization to whole cat allergen extract in a representative population enriched with asthma sample.

## 2 METHODS

### 2.1 Study population

The study participants were recruited from the West Sweden Asthma Study (WSAS), which is a population‐based cohort study.^29^ The procedures and the protocol of the WSAS have been outlined thoroughly elsewhere.^29^, ^30^, ^31^, ^32^ The clinical examination part of the cohort was designed as a population‐based study enriched with asthma sample (Figure S1). Briefly, subjects aged 16–75 years, residing in the Västra Götaland region of western Sweden were randomly recruited and invited to complete a postal survey. Of 30,000 questionnaires distributed, 18,087 replied. A randomly selected sample of 2000 participants was invited for clinical assessment and 1172 individuals agreed to participate. Secondly, 1524 individuals with asthma were invited and 834 of them agreed to participate. Of the total of 2006 participants, 1872 were tested for serum IgE levels against a mix of aeroallergens (Phadiatop^TM^).^32^, ^33^ For the current study, participants who had allergic sensitization to cat dander extract (e1) based on IgE positivity (≥0.35 kU_A_/l) were included (see Figure S1 in the Online Repository), resulting in 361 participants who met the inclusion criteria both originating from random and asthma samples. Additionally, we investigated the baseline features of participants originating from random sample and all participants with asthma (those with asthma originating from both random and asthma sample) (Figure S1). WSAS was approved by the Ethics Committee of the University of Gothenburg (Approval number: 034‐08, 593‐08). All participants gave written informed consent.

### 2.2 Cat molecular allergens

The participants who had allergic sensitization to cat dander (≥0.35 kU_A_/l) were further tested for specific cat molecular allergens (Fel d 1, Fel d 2, Fel d 4, and Fel d 7) using the ImmunoCAP^TM^ system (Phadia AB, Uppsala, Sweden).^32^ IgE levels equal to or more than 0.35 kUA/l were defined as the cut‐off value for the sensitization to molecular allergens.

### 2.3 Sensitization patterns

We defined allergen protein families as follows: lipocalins (Fel d 4 and Fel d 7), albumin (Fel d 2), and secretoglobin (Fel d 1).^4^, ^9^

Sensitization patterns were characterized for cat molecular allergens as below^31^:

(i) *Monosensitization* was defined as sensitization to *only one* cat molecular allergen
(ii) *Polysensitization* was defined as IgE positivity to *three or more* cat molecular allergens^24^
(iii) *Concomitant sensitization* was defined as simultaneous sensitization to more than a single molecular allergen or more than one protein family.

### 2.4 Participant characteristics

Data concerning sex, age, smoking (i.e.: current smoker, non‐smoker, ex‐smoker), body mass index (BMI), urbanization, dust/fume exposure at workplace, education level, having raised in a farm, history of allergy/asthma in family, and cat ownership (childhood and current), presence of current asthma, presence of rhinitis were obtained through the survey and clinical interview.^29^, ^30^, ^32^

Participants who responded positively to either of the following questions: *“Have you ever had asthma?” or “Have you ever been diagnosed as having asthma by a physician?” in combination with any of the following: use of asthma medication, recurrent wheeze, or attacks of shortness of breath during the last* *12 months* was defined as “current asthma”.^31^, ^33^

### 2.5 Statistical analyses

Venn diagrams were used to describe the cosensitization patterns to the cat molecular allergens (“Venndiagram” package, R Foundation for Statistical Computing, Vienna, Austria).^34^ Sensitization patterns to single molecular allergens were also described separately for the following subgroups: (i) presence vs. absence of asthma (ii) presence vs. absence of allergic rhinitis; (iii) presence vs. absence of cat ownership; (iv) males vs. females; (v) obesity (BMI ≥ 30) vs. non‐obesity; and (vi) smoking status. Statistical differences between categorical background variables were analysed using the Pearson Chi‐square test and Fisher's exact test. Since the IgE levels were non‐normally distributed, IgE values were analysed using the Mann‐Whitney *U* test. A two‐sided *p*‐value less than 0.05 was considered statistically significant. Data analyses were implemented using IBM Statistical Package for Social Sciences version 29.0.0.0 (IBM Corporation. Armonk, NY) and GraphPad Prism Version 9.0.0 (GraphPad Software, San Diego, CA).

## 3 RESULTS

### 3.1 Main demographic and clinical characteristics of the study population (*n* = 361)

Among 361 subjects sensitized to whole extracts, 90.9% (*n* = 328) showed IgE positivity to at least one cat molecular allergen, while 9.1% (*n* = 33) tested negative for all measured molecular allergens (Table S1). Thus, 19.3% (361/1872) of the studied population, enriched with asthma sample, was sensitized to cat dander (Figure S1).

### 3.2 Sensitization patterns to cat molecular allergens (*n* = 361)

Fel d 1 was the single molecular allergen to which most subjects sensitized to (84.2%), followed by Fel d 7 (31.3%), Fel d 4 (31.0%), and Fel d 2 (11.9%) (Table S1). Monosensitization to secretoglobin was seen in 45.4% of the subjects, while sensitization to only lipocalins and albumin was less common (3.9% and 1.1%, respectively) in the study population. Polysensitization to 3 or more cat molecular allergens was present in 20.2% of the subjects. On the other hand, concomitant sensitization to all allergen protein families was seen only in 7.2% of subjects. While sensitization to lipocalins differed by age so that subjects older than 60 years were less polysensitized than other age groups, other sensitization patterns did not differ by sex and age groups (Table S1). Eighteen percent of the subjects reported current cat ownership and 30.5% reported cat ownership during childhood (Table S2).

### 3.3 Characteristics and sensitization patterns among subjects sensitized to at least one cat molecular allergen (*n* = 328)

Sensitization patterns were subdivided into three groups according to allergen protein families: lipocalins (Fel d 4 and Fel d 7), albumin (Fel d 2), and secretoglobin (Fel d 1).^4^

Age, sex, smoking status, BMI, dust exposure, farm live, family allergy/asthma history, and cat ownership were demonstrated for the random sample and the asthma sample, including subsamples for those who were sensitized to lipocalins, albumin, and secretoglobin (Table 1). Younger subjects sensitized to secretoglobin were more likely to be from the asthma sample than older subjects (Table 1).

**TABLE 1:** Demographical and clinical characteristics of the random sample and asthma sample according to sensitization patterns among subjects who were sensitized to at least one cat molecular allergen (*n* = 328).

Demographical and clinical characteristics, *n* (%)			Sensitization to lipocalins^a^				Sensitization to albumin^b^				Sensitization to secretoglobin^c^			
			(*n* = 153)^f^				(*n* = 43)^f^				(*n* = 304)^f^			
	Random sample^d^	Asthma sample^e^	Random sample	*p*‐value	Asthma sample	*p*‐value	Random sample	*p‐*value	Asthma sample	*p‐*value	Random sample	*p‐*value	Asthma sample	*p‐*value
	(*n* = 104)	(*n* = 267)	(*n* = 34)		(*n* = 138)		(*n* = 9)		(*n* = 39)		*(n* = 98)		(*n* = 246)	
Sex				0.697				1.000		1.000		0.618		0.677
Males	54 (51.9)	133 (49.8)	15 (20.8)		64 (88.9)	0.608	4 (21.1)		17 (89.5)		52 (33.5)		124 (80.0)	
Females	50 (48.1)	134 (50.2)	19 (23.5)		74 (91.4)		5 (20.8)		22 (91.7)		46 (30.9)		122 (81.9)	
Age, years				0.627		0.060		0.808		0.281		0.093		0.044
≤30	23 (22.1)	64 (24.0)	6 (19.4)		29 (93.5)		1 (11.1)		8 (88.9)		22 (31.0)		59 (83.1)	
31–45	30 (28.8)	102 (38.2)	11 (18.6)		57 (96.6)		3 (20.0)		15 (100.0)		29 (26.9)		93 (86.1)	
46–60	34 (32.7)	77 (28.8)	15 (28.3)		44 (83.0)		4 (25.0)		14 (87.5)		30 (33.0)		72 (79.1)	
61–75	17 (16.3)	24 (9.0)	2 (20.0)		8 (80.8)		1 (33.3)		2 (66.7)		17 (50.0)		22 (64.7)	
Smoking status				0.099		0.172		0.973		0.704		0.511		0.074
Non‐smokers	70 (67.3)	160 (59.9)	23 (25.3)		79 (86.8)		5 (20.0)		22 (88.0)		66 (34.6)		147 (77.0)	
Ex‐smokers	21 (20.2)	68 (25.5)	4 (10.3)		38 (97.4)		3 (21.4)		13 (92.9)		20 (27.4)		64 (87.7)	
Current smokers	13 (12.5)	39 (14.6)	7 (30.4)		21 (91.3)		1 (25.0)		4 (100.0)		12 (30.0)		35 (87.5)	
BMI, kg/m^2^				0.605		0.151		0.547		0.768		0.992		0.599
<25	40 (38.5)	99 (37.1)	10 (17.9)		53 (94.6)		3 (25.0)		11 (91.7)		36 (31.9)		90 (79.6)	
25–29.9	45 (43.3)	116 (43.4)	16 (24.2)		56 (84.8)		6 (22.0)		24 (88.9)		44 (32.6)		108 (80.0)	
≥30	19 (18.3)	52 (19.5)	8 (25.8)		29 (93.5)		0 (0.0)		4 (100.0)		18 (32.1)		48 (85.7)	
Occupational exposure to dust/fumes				0.169		0.523		1.000		1.000		0.147		0.298
No	87 (83.7)	203 (76.0)	29 (24.8)		104 (88.9)		7 (21.2)		30 (90.9)		81 (34.3)		188 (79.7)	
Yes	17 (16.3)	64 (24.0)	5 (13.9)		34 (94.4)		2 (20.2)		9 (90.0)		17 (25.0)		58 (85.3)	
Raise on a farm				0.200		1.000		1.000		1.000		0.931		0.200
No	99 (95.2)	254 (95.1)	34 (23.4)		130 (89.7)		9 (22.5)		36 (90.0)		93 (32.3)		235 (81.6)	
Yes	5 (4.8)	13 (4.9)	0 (0.0)		8 (100.0)		0 (0.0)		3 (100.0)		5 (31.3)		11 (68.8)	
Urbanization degree				0.050		0.392		0.281		0.345		0.067		0.451
>10,000 inhabitants	88 (84.6)	199 (74.5)	28 (26.7)		93 (88.6)		6 (28.6)		18 (85.7)		82 (34.9)		188 (80.0)	
≤10,000 inhabitants	16 (15.4)	68 (25.5)	6 (12.5)		45 (93.8)		3 (13.6)		21 (95.5)		16 (23.2)		58 (84.1)	
Highest education level				0.022		0.315		0.311		0.445		0.026		0.266
Less than high school	9 (8.7)	33 (12.4)	1 (5.9)		17 (100.0)		0 (0.0)		6 (100.0)		9 (27.3)		28 (84.8)	
High school	33 (31.7)	111 (41.6)	12 (16.9)		64 (90.1)		4 (20.0)		17 (85.0)		30 (24.6)		103 (84.4)	
Tertiary	62 (59.6)	123 (46.1)	21 (32.3)		57 (87.7)		5 (29.4)		16 (94.1)		59 (39.6)		115 (77.2)	
Family history of allergy or asthma				0.965		0.661		0.281		1.000		0.707		1.000
No	37 (35.6)	93 (34.8)	13 (22.0)		54 (91.5)		6 (28.6)		19 (90.5)		34 (33.7)		82 (81.2)	
Yes	67 (64.4)	174 (65.2)	21 (22.3)		84 (89.4)		3 (13.6)		20 (90.9)		64 (31.5)		164 (80.8)	
Current cat ownership				0.875		0.741		0.446		1.000		0.564		0.496
No	90 (86.5)	227 (85.0)	27 (22.5)		109 (90.8)		7 (25.9)		24 (88.9)		87 (32.8)		216 (81.5)	
Yes	14 (13.5)	40 (15.0)	7 (21.2)		29 (87.9)		2 (12.5)		15 (93.8)		11 (28.2)		30 (76.9)	
Cat ownership during childhood				0.222		0.332		0.457		0.108		0.229		0.944
No	76 (73.1)	191 (71.5)	19 (19.2)		91 (91.9)		3 (14.3)		21 (100.0)		75 (34.2)		177 (80.8)	
Yes	28 (26.9)	76 (28.5)	15 (27.8)		47 (87.0)		6 (27.3)		18 (81.8)		23 (27.1)		69 (81.2)	
Presence of current allergic rhinitis				0.359		0.753		1.000		0.267		0.010		0.006
No	34 (32.7)	56 (21.0)	6 (16.7)		32 (88.9)		2 (18.2)		9 (81.8)		31 (44.9)		48 (69.6)	
Yes	70 (67.3)	211 (79.0)	28 (23.9)		106 (90.6)		7 (21.9)		30 (93.8)		67 (28.5)		198 (84.3)	

^a^ All participants with sensitization to lipocalins (Fel d 4 or Fel d 7).

^b^ All participants with sensitization to albumin (Fel d 2).

^c^ All participants with sensitization to secretoglobin (Fel d 1).

^d^ Participants originating from the random sample (*n* = 104) consisted of subjects both with asthma (*n* = 43) and without asthma (*n* = 61) that were randomly selected from survey respondents. The given percentages were computed within each group (according to columns).

^e^ Asthma sample (*n* = 267) consists of all subjects with asthma both originating from random (*n* = 43) and asthma sample (*n* = 224). Therefore, patients with asthma originating from the random sample (*n* = 43) were included in both groups. The given percentages were computed within each group (according to columns).

^f^ The given percentages were computed within each independent variable (according to rows) for the comparison of background characteristics. Study groups are not mutually exclusive since one participant could display IgE positivity to different cat molecular allergens at the same time. One particular participant can be included in different groups.

Among individuals with sensitization to at least one cat molecular allergen (*n* = 328), most were sensitized to the secretoglobin family (92.7%), followed by lipocalins (46.6%) and albumin (13.1%) (Table 2).

**TABLE 2:** Demographical and clinical characteristics according to the sensitization patterns of participants who were sensitized to at least one cat molecular allergen (*n* = 328).

Demographical and clinical characteristics, *n* (%)	Sensitized to at least one cat molecular allergen	Sensitization to lipocalins^a^	*p*‐value	Sensitization to albumin^b^	*p*‐value	Sensitization to secretoglobin^c^	*p*‐value
	*N* = 328	*n* = 153 (46.6%)		*n* = 43 (13.1%)		*n =* 304 (92.7%)	
	*n* (%)^d^	*n* (%)^e^		*n* (%)^e^		*n* (%)^e^	
Sex			0.272		0.389		0.379
Males	165 (50.3)	72 (43.6)		19 (11.5)		155 (93.9)	
Females	163 (49.7)	81 (49.7)		24 (14.7)		149 (91.4)	
Age, years			0.033		0.649		0.953
≤30	76 (23.2)	31 (40.8)		9 (11.8)		71 (93.4)	
31–45	117 (35.7)	59 (50.4)		15 (12.8)		108 (92.3)	
46–60	99 (30.2)	53 (53.5)		16 (16.2)		91 (91.9)	
61–75	36 (11.0)	10 (27.8)		3 (8.3)		34 (94.4)	
Smoking status			0.568		0.293		0.571
Non‐smokers	205 (62.5)	91 (44.4)		25 (12.2)		191 (93.2)	
Ex‐smokers	78 (23.8)	39 (50.0)		14 (17.9)		73 (93.6)	
Current smokers	45 (13.7)	23 (51.1)		4 (8.9)		40 (88.9)	
BMI, kg/m^2^			0.685		0.024		0.697
<25	124 (37.8)	56 (45.2)		12 (9.7)		113 (91.1)	
25–29.9	144 (43.9)	66 (45.8)		27 (18.8)		135 (93.8)	
≥30	60 (18.3)	31 (51.7)		4 (6.7)		56 (93.3)	
Occupational exposure to dust/fumes			0.695		0.907		0.767
No	254 (77.4)	117 (46.1)		33 (13.0)		236 (92.9)	
Yes	74 (22.6)	36 (48.6)		10 (13.5)		68 (91.9)	
Raised on a farm			0.847		0.716		0.631
No	310 (94.5)	145 (46.8)		40 (12.9)		288 (92.9)	
Yes	18 (5.5)	8 (44.4)		3 (16.7)		16 (88.9)	
Urbanization degree			0.004		<0.001		0.036
>10,000 inhabitants	249 (75.9)	105 (42.2)		21 (8.4)		235 (94.4)	
≤10,000 inhabitants	79 (24.1)	48 (60.8)		22 (27.8)		69 (87.3)	
Highest education level			0.096		0.475		0.281
Less than high school	38 (11.6)	17 (44.7)		6 (15.8)		33 (86.8)	
High school	132 (40.2)	71 (53.8)		20 (15.2)		122 (92.4)	
Tertiary	158 (48.2)	65 (41.1)		17 (10.8)		149 (94.3)	
Family history of allergy or asthma			0.176		0.038		0.038
No	114 (34.8)	59 (51.8)		21 (18.4)		101 (88.6)	
Yes	214 (65.2)	94 (43.9)		22 (10.3)		203 (94.9)	
Current cat ownership			0.005		<0.001		<0.001
No	277 (84.5)	120 (43.3)		27 (9.7)		265 (95.7)	
Yes	51 (15.5)	33 (64.7)		16 (31.4)		39 (76.5)	
Cat ownership during childhood			0.018		<0.001		0.154
No	233 (71.0)	99 (42.5)		21 (9.0)		219 (94.0)	
Yes	95 (29.0)	54 (56.8)		22 (23.2)		85 (89.5)	
Presence of current asthma			<0.001		0.003		0.342
No	111 (33.8)	32 (28.8)		6 (5.4)		105 (94.6)	
Yes	217 (66.2)	121 (55.8)		37 (17.1)		199 (91.7)	
Presence of current allergic rhinitis			0.826		0.806		0.036
No	79 (24.1)	36 (45.6)		11 (13.9)		69 (87.3)	
Yes	249 (75.9)	117 (47.0)		32 (12.9)		235 (94.4)	

^a^ All participants with sensitization to lipocalins (Fel d 4 or Fel d 7).

^b^ All participants with sensitization to albumin (Fel d 2).

^c^ All participants with sensitization to secretoglobin (Fel d 1).

^d^ The given percentages were computed within each group (according to columns).

^e^ The given percentages were computed within each independent variable (according to rows). Study groups are not mutually exclusive since one participant could display IgE positivity to different cat molecular allergens at the same time. One particular participant can be included in different groups.

Subjects with current cat ownership were more likely to be sensitized to lipocalins (64.7% vs. 43.3%, *p* = 0.005) and albumin (31.4% vs. 9.7%, *p* < 0.001) than those not owning a cat. Likewise, those who owned a cat during childhood were more frequently sensitized to lipocalins (56.8% vs. 42.5%, *p* = 0.018) and albumin (23.2% vs. 9.0%, *p* < 0.001) than those who did not own a cat during childhood (Table 2). On the other hand, current cat owners were significantly less sensitized to secretoglobin than non‐cat owners (76.5% vs. 95.7%, *p* < 0.001), but it did not show any difference for cat owners during childhood (Table 2).

Sensitization to protein allergen families also differed according to the degree of urbanization (Table 2). Those living in densely populated urban areas were less likely to be sensitized to lipocalins and albumin than those living in sparsely populated areas (Table 2). On the other hand, subjects residing in densely populated urban areas were more likely to be sensitized to secretoglobin than those residing in sparsely populated areas (94.4% vs. 87.3%, *p* = 0.036). Additionally, subjects who had a family history of allergy or asthma were more commonly sensitized to secretoglobin compared to those without, while there was no difference in terms of sensitization to lipocalins (Table 2).

Subjects with asthma were more likely to be sensitized to lipocalins (55.8% vs. 28.8%, *p* < 0.001) and albumin (17.1% vs. 5.4%, *p* = 0.003), but not to secretoglobin (91.7% vs. 94.6%, *p* = 0.342) than those without asthma (Table 2).

Table 3 shows the distribution of background characteristics by monosensitization and concomitant sensitization to all protein allergen families. Monosensitization to secretoglobin was more common among the youngest and oldest age groups than those in the middle ages. In addition, monosensitization to secretoglobin was also more common among those residing in densely populated areas than in sparsely populated areas, and among those without than those who currently own or owned a cat during childhood (Table 3).

**TABLE 3:** Demographical and clinical characteristics according to the monosensitization, concomitant sensitization to all allergen protein families, and polysensitization status among subjects who were sensitized to at least one cat molecular allergen (*n* = 328).

Demographical and clinical characteristics, *n* (%)	Sensitization to only lipocalins^a^	*p*‐value	Monosensitization to albumin^b^	*p*‐value	Monosensitization to secretoglobin^c^	*p*‐value	Concomitant sensitization^d^	*p*‐value	Polysensitization^e^	*p*‐value
	*n* = 14 (4.3%)^f^		*n* = 4 (1.2%)^f^		*n* = 164 (50.0%)^f^		*n* = 26 (7.9%)^f^		*n* = 73 (22.3%)^f^	
Sex		0.981		1.000		0.320		0.659		0.941
Males	7 (4.2)		2 (1.2)		87 (52.7)		12 (7.3)		37 (22.4)	
Females	7 (4.3)		2 (1.2)		77 (47.2)		14 (8.6)		36 (22.1)	
Age, years		0.932		0.869		0.031		0.319		0.088
≤30	3 (3.9)		1 (1.3)		44 (57.9)		7 (9.2)		18 (23.7)	
31–45	6 (5.1)		2 (1.7)		54 (46.2)		10 (8.5)		29 (24.8)	
46–60	4 (4.0)		1 (1.0)		42 (42.4)		9 (9.1)		24 (24.2)	
61–75	1 (2.8)		0 (0.0)		24 (66.7)		0 (0.0)		2 (5.6)	
Smoking status		0.693		0.297		0.697		0.325		0.592
Non‐smokers	8 (3.9)		4 (2.0)		106 (51.7)		15 (7.3)		45 (22.0)	
Ex‐smokers	3 (3.8)		0 (0.0)		36 (46.2)		9 (11.5)		20 (25.6)	
Current smokers	3 (6.7)		0 (0.0)		22 (48.9)		2 (4.4)		8 (17.8)	
BMI, kg/m^2^		0.488		0.745		0.746		0.166		0.610
<25	7 (5.6)		2 (1.6)		65 (52.4)		7 (5.6)		25 (20.2)	
25–29.9	4 (2.8)		1 (0.7)		71 (49.3)		16 (11.1)		32 (22.2)	
≥30	3 (5.0)		1 (1.7)		28 (46.7)		3 (5.0)		16 (26.7)	
Occupational exposure to dust/fumes		1.000		0.220		0.597		0.948		0.881
No	11 (4.3)		2 (0.8)		129 (50.8)		20 (7.9)		57 (22.4)	
Yes	3 (4.1)		2 (2.7)		35 (47.3)		6 (8.1)		16 (21.6)	
Raise on a farm		1.000		1.000		0.628		1.000		0.088
No	14 (4.5)		4 (1.3)		154 (49.7)		25 (8.1)		72 (23.2)	
Yes	0 (0.0)		0 (0.0)		10 (55.6)		1 (5.6)		1 (5.6)	
Urbanization degree		0.750		1.000		0.003		<0.001		0.009
>10,000 inhabitants	10 (4.0)		3 (1.2)		136 (54.6)		12 (4.8)		47 (18.9)	
≤10,000 inhabitants	5 (5.1)		1 (1.3)		28 (35.4)		14 (17.7)		26 (32.9)	
Highest education level		0.423		0.753		0.079		0.350		0.033
Less than high school	3 (7.9)		0 (0.0)		21 (55.3)		4 (10.5)		6 (15.8)	
High school	6 (4.5)		2 (1.5)		56 (42.4)		13 (9.8)		39 (29.5)	
Tertiary	5 (3.2)		2 (1.3)		87 (55.1)		9 (5.7)		28 (17.7)	
Family history of allergy or asthma		0.023		0.612		0.064		0.089		0.508
No	9 (7.9)		2 (1.8)		49 (43.0)		13 (11.4)		23 (20.2)	
Yes	5 (2.3)		2 (0.9)		115 (53.7)		13 (6.1)		50 (23.4)	
Current cat ownership		0.002		0.116		<0.001		0.010		0.015
No	7 (2.5)		2 (0.7)		150 (54.2)		17 (6.1)		55 (19.9)	
Yes	7 (13.7)		2 (3.9)		14 (27.5)		9 (17.6)		18 (35.3)	
Cat ownership during childhood		1.000		0.582		0.002		0.044		0.021
No	10 (4.3)		2 (0.9)		129 (55.4)		14 (6.0)		44 (18.9)	
Yes	4 (4.2)		2 (2.1)		35 (36.8)		12 (12.6)		29 (30.5)	
Presence of current asthma		0.397		0.114		<0.001		0.003		<0.001
No	3 (2.7)		3 (2.7)		75 (67.6)		2 (1.8)		11 (9.9)	
Yes	11 (5.1)		1 (0.5)		89 (41.0)		24 (11.1)		62 (28.6)	
Presence of current allergic rhinitis		0.750		0.045		0.698		0.119		0.623
No	4 (5.1)		3 (3.8)		38 (48.1)		3 (3.8)		16 (20.3)	
Yes	10 (4.0)		1 (0.4)		126 (50.6)		23 (9.2)		57 (22.9)	

^a^ Participants with only sensitization to any lipocalin (Fel d 4 or Fel d 7).

^b^ Participants with only sensitization to albumin (Fel d 2).

^c^ Participants with only sensitization to secretoglobin (Fel d 1).

^d^ Concomitant sensitization to all allergen protein families (secretoglobin. albumin, and any lipocalin).

^e^ Sensitized to 3 or more of the cat molecular allergens.

^f^ The given percentages were computed within each independent variable (according to rows).

Sensitization to only lipocalins was more common among those who currently owned a cat than non‐current cat owners (13.7% vs. 2.5%, *p* = 0.002) and also among those without than those with a family history of allergy or asthma (7.9% vs. 2.3%, *p* = 0.023). Moreover, sensitization to only lipocalins was similar between subjects with and without asthma, while monosensitization to secretoglobin was less common in subjects with asthma than those without (Table 3). Comparison of subjects who were monosensitized to secretoglobin and sensitized to only lipocalins was also presented in Table S3.

Concomitant sensitization to all protein families was more common among those residing in less densely populated areas than in more densely populated areas (17.7% vs. 4.8%, *p* < 0.001); among those with current cat ownership than those without (17.6% vs. 6.1%, *p* = 0.010); and among those with asthma than those without (11.1% vs. 1.8%, *p* = 0.003). Finally, polysensitization was more common among those residing in sparsely populated areas than in densely populated areas (32.9% vs. 18.9%, *p* = 0.009); among those who currently own or owned a cat during childhood than those without; and among those with asthma than those without (28.6% vs. 9.9%, *p* < 0.001) (Table 3).

### 3.4 Concomitant sensitization to single cat molecular allergens among subjects sensitized to at least one cat molecular allergen (*n* = 328)

**FIGURE 1:**
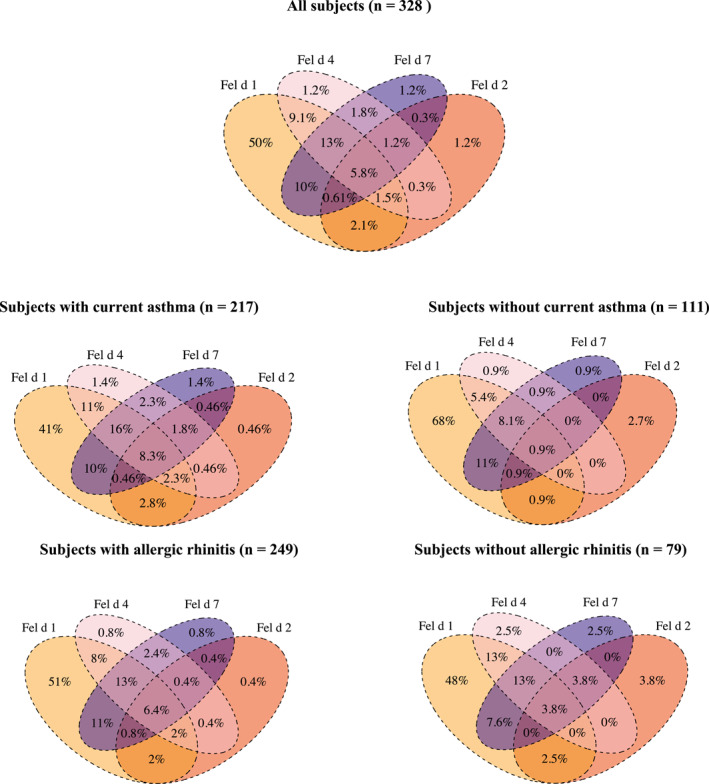
Venn diagram for sensitization patterns to cat molecular allergens among subjects sensitized to at least one cat molecular allergen, subjects with and without current asthma, subjects with and without allergic rhinitis.

The most common concomitant sensitization pattern amongst the cat molecular allergens was between Fel d 1 and Fel d 7 (*n* = 98, 30%) (Figure 1). Concomitant sensitization to single allergens was more common in subjects with asthma compared with those without asthma in every case (Figure 1). Among subjects with asthma, the most common overlapping pair was concomitant sensitization to Fel d 1 and Fel d 4 (37%), while concomitant sensitization to Fel d 1 and Fel d 7 (32%) was the most common overlap in subjects with allergic rhinitis (Figure 1).

Sensitization was different between current and non‐current cat owners and between those who owned a cat during childhood and those who did not own a cat (Figure S2). Concomitant sensitization to Fel d 4 and Fel d 7 (39%) was the most common overlap among current cat owners, while it was between Fel d 1 and Fel d 4 (39%) among cat owners during childhood (Figure S2). Concomitant sensitization to single allergens did not differ statistically according to sex, obesity, and smoking (Figures S3–S5).

### 3.5 IgE levels to cat dander and cat molecular allergens among subjects sensitized to at least one cat molecular allergen (*n* = 328)

**FIGURE 2:**
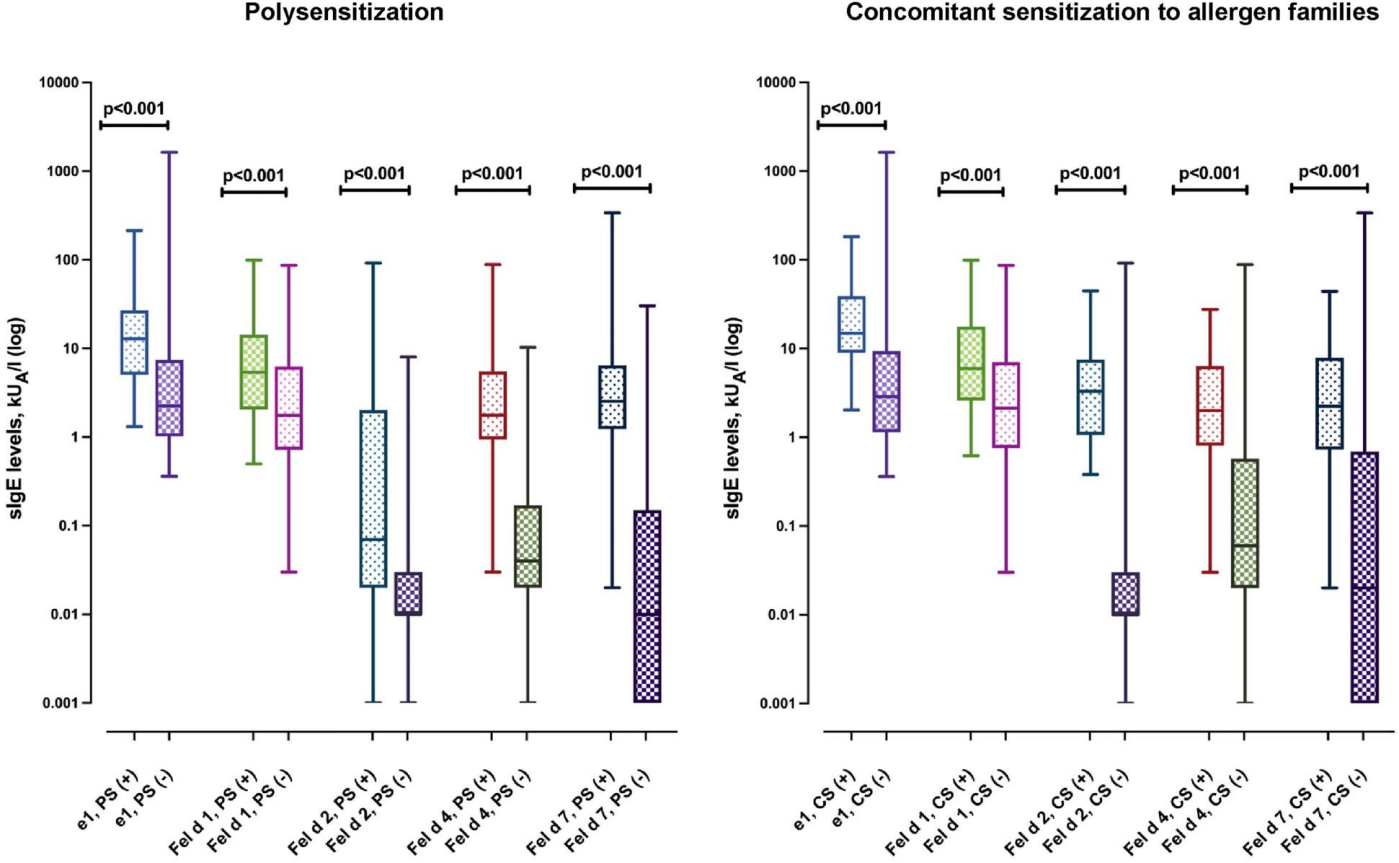
sIgE levels to cat molecular allergens according to polysensitization, and concomitant sensitization to all protein allergen families (lipocalins, albumin, and secretoglobin). Data are presented as median, maximum, and minimum values (whiskers). IgE levels were compared using the Mann‐Whitney *U* test. CS, concomitant sensitization; e1, cat dander immunoglobulin E; PS, polysensitization; sIgE, specific immunoglobulin E.

IgE levels to each allergen were significantly higher in polysensitized subjects compared with those without polysensitization (Figure 2). Similarly, subjects with concomitant sensitization to all protein allergen families also had increased IgE levels against each cat molecular allergen than those without concomitant sensitization. In addition, IgE levels to cat dander extract were significantly higher in subjects with poly‐ and concomitant sensitization to all protein families than in those without (Figure 2).

**FIGURE 3:**
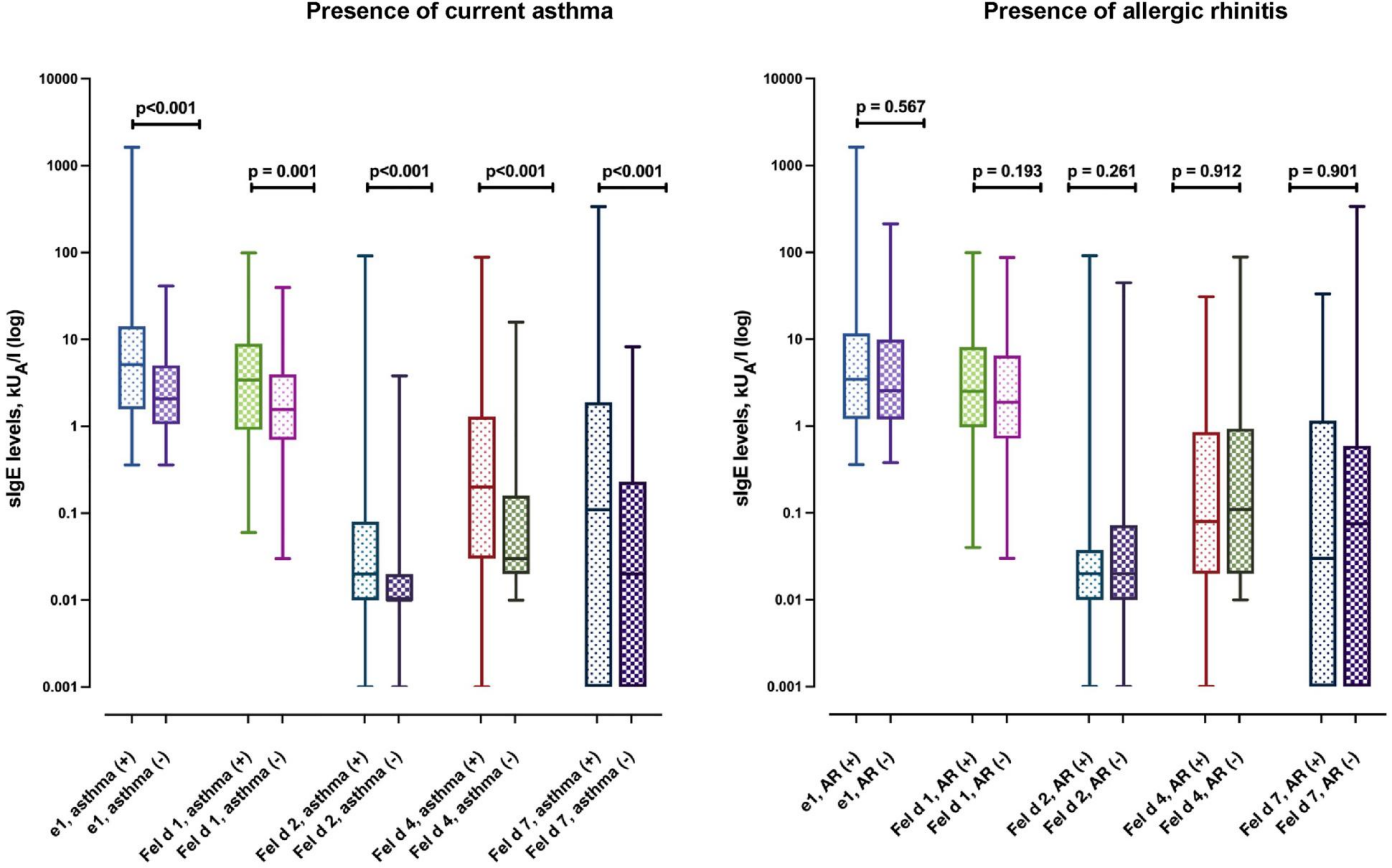
sIgE levels to cat molecular allergens in the presence of current asthma and allergic rhinitis. Data are presented as median, maximum, and minimum values (whiskers). IgE levels were compared using the Mann‐Whitney *U* test. AR, allergic rhinitis; e1, cat dander immunoglobulin E; sIgE, specific immunoglobulin E.

Subjects with asthma had increased IgE levels against whole cat extract than subjects without asthma (5.15 kU_A_/l vs. 2.08 kU_A_/l, respectively, *p* < 0.001), while it did not differ according to the presence of allergic rhinitis (3.46 kU_A_/l vs. 2.56 kU_A_/l, respectively, *p* = 0.567) (Figure 3). Likewise, IgE levels of each allergen component were significantly higher in subjects with asthma than in those without asthma, but did not differ between those with and without allergic rhinitis (Figure 3). The IgE levels to cat dander extract and molecular allergens also differed according to current cat ownership and cat ownership during childhood (Figure S6). Current cat owners had significantly higher IgE levels to cat dander extract, Fel d 2, and Fel d 4 than those without cats currently, while cat owners during childhood had only higher IgE levels to Fel d 4 (Figure S6).

Lastly, subjects with monosensitization to secretoglobin had significantly lower cat dander IgE levels compared with those without. However, this was not the case for subjects sensitized to only lipocalins (Figure S7).

## 4 DISCUSSION

### 4.1 Summary of key findings

In this population‐based sample of adults enriched with asthma, we found that while the majority of subjects were sensitized to Fel d 1, monosensitization to Fel d 1 was present in half of the subjects. Fel d 1 sensitization did not differ between subjects with and without asthma, while levels of the IgE concentration did. In addition, subjects with asthma were more often polysensitized and had more overlapping sensitization than subjects without asthma. Varying sensitization patterns between cat molecular allergens also showed differences in subjects' background characteristics. Cat owners and individuals living in rural areas were more likely to be sensitized to lipocalins and albumin as well as a higher degree of polysensitization and concomitant sensitization to protein families, but less likely to be sensitized to secretoglobin compared to those without.

### 4.2 Comparison of findings with the previous literature

Fel d 1 was the major cat allergen molecule and the indicator of primary allergic sensitization to cat in our study, in line with the current literature.^1^, ^9^ Our results also confirm the findings of Grönlund et al that showed that Fel d 1 is a good marker for cat allergy, although, half of the subjects were not monosensitized to Fel d 1.^22^ Measurement of minor allergens, in addition to major cat allergen, Fel d 1, could help to identify different phenotypes of cat sensitization and also give additional information about the clinical course.^1^, ^35^, ^36^

The contribution of this study is to elaborate on the patterns and prevalence of sensitization to specific cat molecular allergens in a representative adult population enriched with asthma sample. Our data show that subjects with cat sensitization do not have homogenous features but show distinct differences in their sensitization profiles. Polysensitization and concomitant sensitization to all protein allergen families were associated with asthma prevalence, cat ownership, and cat exposure during childhood and rural living. In addition to increased recognition of IgE molecules, poly‐ and concomitantly sensitized subjects had significantly increased IgE levels to all cat molecular allergens in our study. Likewise, in the BAMSE/MeDALL study, polysensitization to cat/dog allergens was associated with higher IgE levels to molecular allergens in children.^24^ We also showed that poly‐ and concomitantly sensitized subjects had higher IgE levels to cat dander, whereas subjects with monosensitization to secretoglobin had lower IgE levels to whole extract. This indicates that subjects with higher IgE levels to whole extract should be taken into consideration for polysensitization risk. Our findings confirm previous results showing that adults with polysensitization and concomitant sensitization to allergen families had different background and disease features compared to those without.^24^ Identifying the subjects with polysensitization could help to provide a better insight regarding disease outcomes and future risks.

To date, allergens are generally classified according to the frequency of IgE‐binding subjects.^35^ A recent study, which studied all registered cat allergens (Fel d 1 to Fel d 8), found that sensitization to Fel d 3, Fel d 4, and Fel d 7 was observed in more than half of the subjects, hence they suggested to appraise these allergens as major/mid‐tier allergens.^36^ In our data, nearly 30% of the subjects were sensitized to Fel d 4 and Fel d 7. On the other hand, it has been suggested that revealing allergenic activity might be more valuable than defining the allergens based on only the frequency of IgE recognition for clinical implications.^35^ Our results also showed that subjects with asthma had higher frequencies of sensitization to lipocalins and albumin but not to secretoglobin, Fel d 1. Supportingly, in a previous study, IgE reactivity to Fel d 2, Fel d 4, and Fel d 7 were associated with type‐2 inflammation markers; however, no association was found between Fel d 1 sensitization and type‐2 biomarkers in subjects with asthma.^37^ In this context, Caraballo et al. suggested the use of the term “allergens” instead of the current classification based on the frequency since allergenic activity might not always be related to the frequency of IgE‐binding.^35^ Since our results support the idea that sensitization to minor allergens is related to the presence of asthma, a classification reflecting the allergenic activity could improve clinical implications rather than a classification based solely on frequency.^35^

While the relationship between cat ownership and allergic sensitization remains controversial in the current literature, childhood exposure to cats seems to be associated with a complex sensitization pattern in adulthood.^38^ So far, only a few studies have been able to focus on this research topic. Nagao et al. also showed that young children started to develop sensitization to cat allergenic molecules even before developing respiratory‐allergic symptoms.^39^ In our findings, current cat owners were more likely to be sensitized to lipocalins and albumins, but less likely to be sensitized to secretoglobin compared with non‐owners. In a previous study, Hemmer et al. showed that the primary sensitization to Fel d 2, Fel d 4, and Fel d 7 was also higher in cat owners than in non‐cat owners, while this was not the case for sensitization to Fel d 1.^40^ This difference might be affected by the exposure source of allergens.^9^ A previous study showed that Fel d 1 levels were higher compared to Fel d 4 in cat fur, whereas Fel d 4 levels were higher than Fel d 1 in cat saliva on the contrary.^41^ Similarly, Fel d 7 was mainly available in saliva; therefore, increased sensitization rates to lipocalins in cat owners could be explained by closer contact.^9^

Furthermore, subjects who lived in densely populated urban areas were more likely to be sensitized to secretoglobin, Fel d 1, but less likely to be sensitized to lipocalins and albumin than those living in sparsely populated areas. It could be hypothesized that subjects in densely populated areas could be exposed to main cat allergens via secondhand transmission since Fel d 1 is ubiquitously present in public places.^1^, ^9^, ^38^ Therefore, Fel d 1 exposure could occur via secondhand transmission compared to other allergens and could be more related to the number of cats in the community than individual ownership.^1^, ^38^

Interestingly, in a previous study conducted by the MeDALL consortium, sensitization to secretoglobin was positively associated with air pollution, which might be another confounding factor.^42^ On the other hand, subjects who had a family history of allergy/asthma were more likely to be sensitized to secretoglobin, Fel d 1, in our sample. This subgroup might be more likely to avoid pet ownership due to a family history of allergy than subjects without a family history. In a recent review, Moustaki et al suggested that the development of allergic sensitization has been affected by various factors, including environmental factors, time and amount of exposure, and genetic susceptibility, in addition to allergen exposure.^38^ Thus, several predisposing factors should be taken into consideration in the development of allergy.

### 4.3 Strengths and limitations

Given the paucity of previous research in adults, the current study, the first of its kind, advances our understanding of cat allergy at the molecular level and provides an impetus for further studies in cat‐sensitized adults. We included all the commercially available and clinically relevant cat molecular allergens, giving the current study a comprehensive picture of the profiles of cat molecular allergens in an asthma enriched adult sample. On the other hand, allergic sensitization towards cat dander and molecular allergens measured via IgE positivity does not necessarily translate into clinical allergy; thus, clinical cat allergy itself may require further validation with clinical evaluation.^4^ Nevertheless, IgE positivity has been demonstrated and is widely used as an indicative marker for future allergy risk and allergic airway inflammation in epidemiologic studies.^24^, ^43^ Lastly, cross‐reactivity patterns to cat and horse allergens should be taken into account in future studies since it has been commonly described in subjects with allergy to furry animals.^4^

### 4.4 Clinical and research implications of the findings

Our findings indicate that the majority of subjects with sensitization to cat dander extract also tested positive for Fel d 1. Given that Fel d 1 is a species‐specific cat allergen and a sensitive marker for cat allergy, the necessity of performing further analyses for minor cat allergen molecules to improve diagnostic and clinical utility remains a future research question.^1^, ^22^ Considering the high cross‐ and co‐sensitization in furry animals, the measurement of minor allergens could help to identify possible cross‐ and co‐sensitization against other furry animals in subjects sensitized to cat dander.^4^ In addition to increasing diagnostic accuracy, the measurement of the minor cat allergens could help to identify subjects with different sensitization patterns, including poly‐ and concomitant sensitization and understand diverging disease outcomes related to different sensitization patterns.

## 5 CONCLUSION

Among available and clinically relevant cat molecular allergens, sensitization to Fel d 1 is the most common in adults, followed by Fel d 7 and Fel d 4. Polysensitization and concomitant sensitization to all protein allergen families were linked to the presence of asthma, cat exposure, rural living, and elevated IgE levels to cat molecular allergens. Revealing different sensitization profiles and disease phenotypes, CRD could help clinicians to create patient‐tailored risk profiles. Future studies should focus on illness‐related outcomes of asthma and other allergic diseases in large populations.

## AUTHOR CONTRIBUTIONS

Conceptualized the project (Saliha Selin Özuygur Ermis, Aram Norouzi, Hannu Kankaanranta, Linda Ekerljung, Jan Lötvall, Bright I. Nwaru). Responsible for the database (Rani Basna, Linda Ekerljung, Hannu Kankaanranta, Bright I. Nwaru). Participated in data collection (Linda Ekerljung, Madeleine Rådinger, Carina Malmhäll, Jan Lötvall, Hannu Kankaanranta, Bright I. Nwaru). Analysed the data (Saliha Selin Özuygur Ermis, Aram Norouzi, Hannu Kankaanranta, Bright I. Nwaru). Drafted the manuscript (Saliha Selin Özuygur Ermis, Aram Norouzi, Hannu Kankaanranta, Bright I. Nwaru). All authors were involved in data interpretation and confirmed the final version of the submitted manuscript.

## CONFLICT OF INTEREST STATEMENT

Saliha Selin Özuygur Ermis reports conference attendance‐related costs from Thermo Fisher Scientific. Magnus P. Borres is employed by Thermo Fisher Scientific (Uppsala, Sweden). Jan Lötvall and Bright I. Nwaru obtained research materials for the IgE measurements of this work from Thermo Fisher Scientific on behalf of WSAS. Hannu Kankaanranta reports fees for consultancies and lectures from AstraZeneca, Boehringer‐Ingelheim, Chiesi Pharma, GSK, MSD, Novartis, Orion Pharma and Sanofi Genzyme outside the current study. The rest of the authors declare no conflicts of interest associated with this work.

## Supporting information

Supporting Information S1

Supporting Information S2

## Data Availability

Data available on request due to privacy/ethical restrictions.
