# Supplementary material for: Sensitization patterns to cat molecular allergens in subjects with allergic sensitization to cat dander
Source: Clin Transl Allergy. 2023 Aug 12;13(8):e12294. doi: 10.1002/clt2.12294 (PMC10422092; doi:10.1002/clt2.12294)
Supplement: Supplementary file 1 — Supporting Information S1 [file CLT2-13-e12294-s002.docx]

**Figure S1.** Flowchart of the study population.

^†^Participants originating from random sample (n = 104) consists of subjects both with asthma (n = 43) and without asthma (n = 61) that were randomly selected from survey responders.

^‡^All participants with asthma sample (n = 267) consists of all subjects with asthma both originating from random (n = 43) and asthma sample (n = 224). Therefore, patients with asthma originating from random sample included in both groups.

**Current cat owners (n = 51) Non-current cat owners (n = 277)**

**Owned cat during childhood (n = 95) Owned no cat during childhood ( n = 233)**

**Figure S2:** Venn diagram for sensitization patterns to cat molecular allergens among current cat owners vs. no current cat owners, and subjects who owned a cat during childhood vs. those who did not own a cat during childhood.

**Females (n = 163)** **Males (n= 165)**

**Figure S3.** Venn diagram for sensitization patterns to cat molecular allergens among females and males.

**Obese (n = 60) Non-obese (n = 268)**

**Figure S4.** Venn diagram for sensitization patterns to cat molecular allergens in obese and non-obese participants.

**Non-smokers (n = 205 ) Ex-smokers (n = 78)**

**Current smokers ( n = 45)**

**Figure S5.** Venn diagram for sensitization patterns to cat molecular allergens among non-smokers, ex-smokers, and current smoker.

**Current cat ownership Cat ownership during childhood**

**
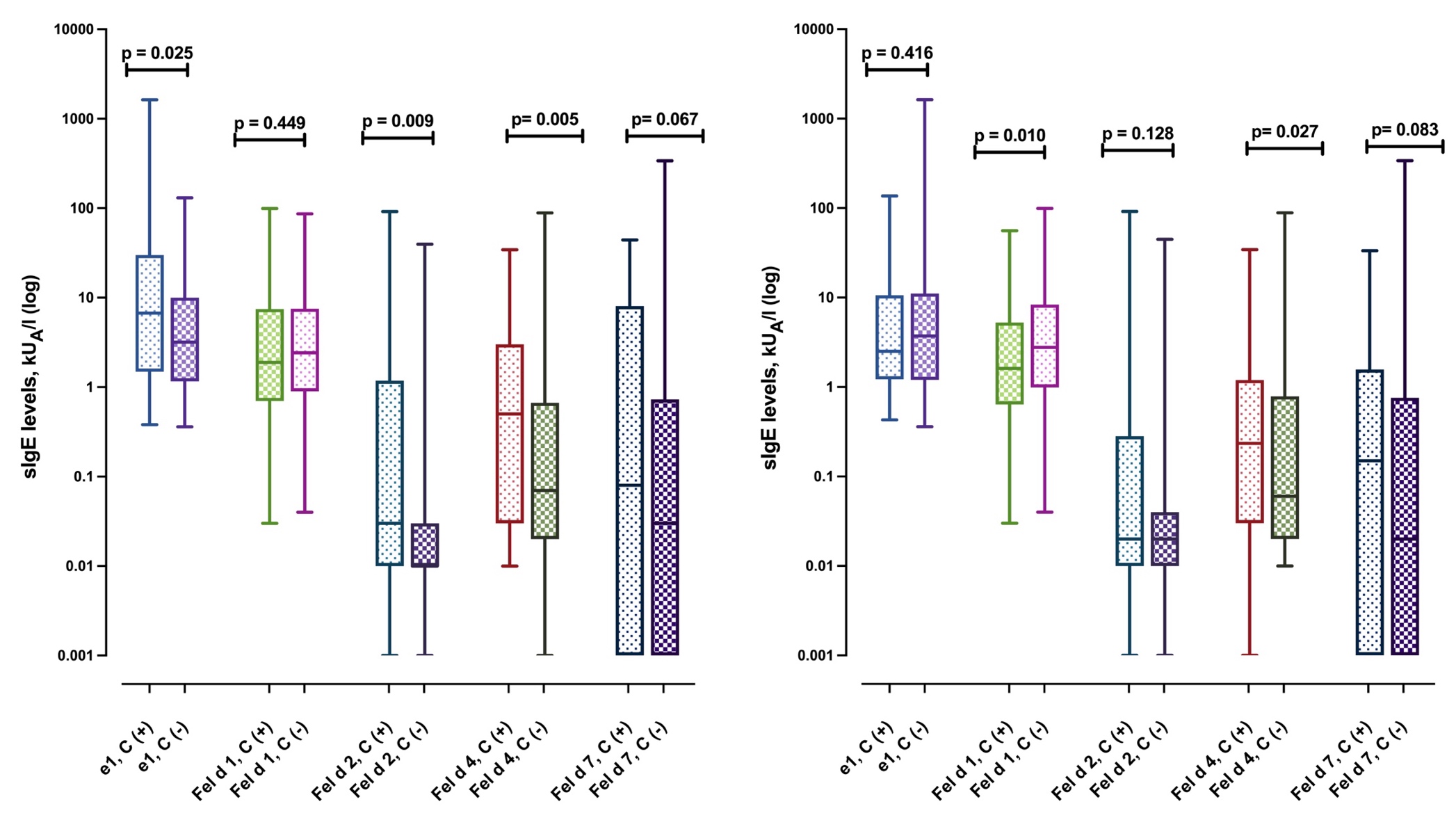
**

**Figure S6:** sIgE levels to cat allergen components by current cat ownership and cat ownership during childhood. C= cat ownership, e1= cat dander immunoglobulin E, sIgE= specific immunoglobin E. Data are presented as median, maximum, and minimum values (whiskers). IgE levels are compared by the Mann-Whitney U test.

**Sensitization to only lipocalins Monosensitization to secretoglobin**

**
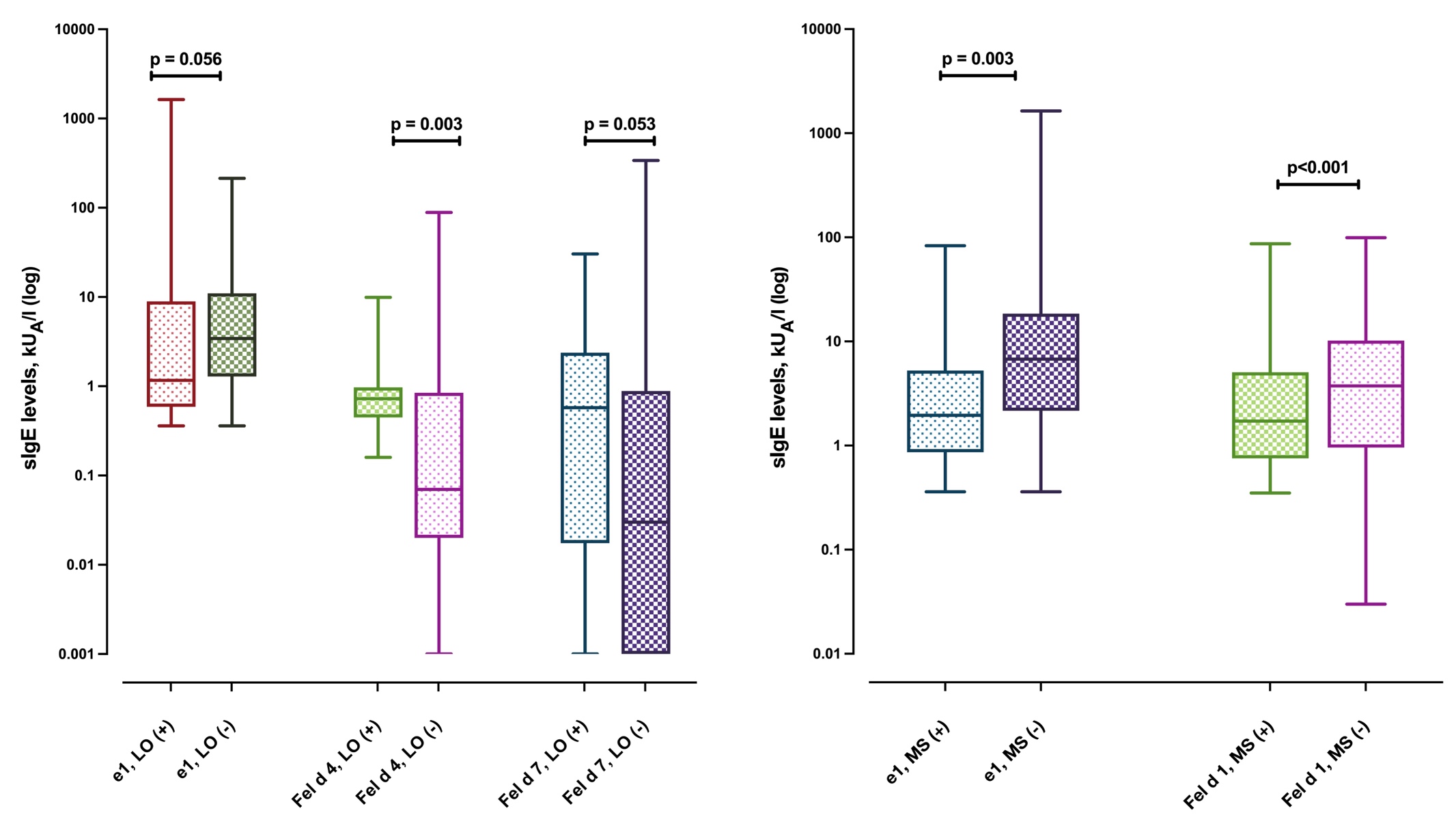
**

**Figure S7.** sIgE levels to cat molecular allergens according to sensitization to only lipocalins (Fel d 4 or Fel d 7) and monosensitization to secretoglobin (Fel d 1). e1= cat dander immunoglobulin E, LO=sensitization to only lipocalins, MS= monosensitization to secretoglobin, sIgE= specific immunoglobin E. Data are presented as median, maximum, and minimum values (whiskers). IgE levels are compared by the Mann-Whitney U test.
