# Supplementary material for: Sensitization patterns to cat molecular allergens in subjects with allergic sensitization to cat dander
Source: Clin Transl Allergy. 2023 Aug 12;13(8):e12294. doi: 10.1002/clt2.12294 (PMC10422092; doi:10.1002/clt2.12294)
Supplement: Supplementary file 2 — Supporting Information S2 [file CLT2-13-e12294-s001.docx]

**Table S1.** Sensitization patterns to cat molecular allergens by age and sex in subjects with sensitization to cat dander extract (n=361)^†^

| **Sensitization patterns, n (%)** | All  *N=361*  *n* (%) | ≤30 years  (*n*= 83) | | 31-45 years  (*n*= 128) | | 46-60 years  (*n*= 107) | | 61-75 years  (*n*= 43) | | P-values for differences | |
| --- | --- | --- | --- | --- | --- | --- | --- | --- | --- | --- | --- |
|  |  | Men  (*n*= 39) | Women  (*n*= 44) | Men  (*n*= 62) | Women  (*n*= 66) | Men  (*n*= 53) | Women  (*n*= 54) | Men  (*n*= 25) | Women  (*n*= 18) | Age | Sex |
| Not sensitized to any cat molecular allergen | 33 (9.1) | 3 (7.7) | 4 (9.1) | 2 (3.2) | 9 (13.6) | 4 (7.5) | 4 (7.4) | 5 (20.0) | 2 (11.1) | 0.378 | 0.388 |
| Sensitized to at least one measured cat molecular allergen | 328 (90.9) | 36 (92.3) | 40 (90.9) | 60 (96.8) | 57 (86.4) | 49 (92.5) | 50 (92.6) | 20 (80.0) | 16 (88.9) | 0.378 | 0.388 |
| Sensitization to lipocalins^1^  Sensitization to Fel d 4  Sensitization to Fel d 7 | 153 (42.4)  112 (31.0)  113 (31.3) | 16 (41.0)  14 (35.9)  11 (28.2) | 15 (34.1)  12 (27.3)  12 (27.3) | 30 (48.4)  24 (38.7)  21 (33.9) | 29 (43.9)  20 (30.3)  23 (34.8) | 22 (41.5)  17 (32.1)  15 (28.3) | 31 (57.4)  19 (35.2)  25 (46.3) | 4 (16.0)  3 (12.0)  3 (12.0) | 6 (33.3)  3 (16.7)  3 (16.7) | 0.016  0.076  0.030 | 0.410  0.575  0.171 |
| Sensitization to serum albumin^2^ | 43 (11.9) | 3 (7.7) | 6 (13.6) | 7 (11.3) | 8 (12.1) | 8 (15.1) | 8 (14.8) | 1 (4.0) | 2 (11.1) | 0.565 | 0.451 |
| Sensitization to secretoglobin^3^ | 304 (84.2) | 34 (87.2) | 37 (84.1) | 54 (87.1) | 54 (81.8) | 47 (88.7) | 44 (81.5) | 20 (80.0) | 14 (77.8) | 0.795 | 0.218 |
| Sensitization to only lipocalins | 14 (3.9) | 2 (5.1) | 1 (2.3) | 3 (4.8) | 3 (4.5) | 2 (3.8) | 2 (3.7) | 0 (0.0) | 1 (5.6) | 0.914 | 0.975 |
| Monosensitization to albumin | 4 (1.1) | 0 (0.0) | 1 (2.3) | 2 (3.2) | 0 (0.0) | 0 (0.0) | 1 (1.9) | 0 (0.0) | 0 (0.0) | 0.859 | 1.000 |
| Monosensitization to secretoglobin | 164 (45.4) | 20 (51.3) | 24 (54.5) | 28 (45.2) | 26 (39.4) | 24 (45.3) | 18 (33.3) | 15 (60.0) | 9 (50.0) | 0.112 | 0.230 |
| Concomitant sensitization to lipocalins, serum albumin, and secretoglobin | 26 (7.2) | 3 (7.7) | 4 (9.1) | 4 (6.5) | 6 (9.1) | 5 (9.4) | 4 (7.4) | 0 (0.0) | 0 (0.0) | 0.280 | 0.716 |
| Polysensitization^4^ | 73 (20.2) | 9 (23.1) | 9 (20.5) | 15 (24.2) | 14 (21.2) | 11 (20.8) | 13 (24.1) | 2 (8.0) | 0 (0.0) | 0.061 | 0.833 |

^1^All participants with sensitization to lipocalins (Fel d 4 or Fel d 7)

^2^All participants with sensitization to albumin (Fel d 2)

^3^All participants with sensitization to secretoglobin (Fel d 1)

^4^Sensitization to 3 or more of the cat molecular allergen

†The given percentages were computed within each group (according to columns). Study groups are not mutually exclusive since one participant could be sensitized to different cat molecular allergens at the same time. Missing data was available for Fel d 2 (n=3), Fel d 4 (n=3), Fel d 7(n= 3), Fel d 1 (n= 9).

**Table S2:** The demographical and clinical characteristics of the study participants (n = 361)

| **Demographical and clinical characteristics** | Frequency  *N* = 361 |
| --- | --- |
| Sex, n (%)  Males  Females | 179 (49.6)  182 (50.4) |
| Age, years, n (%)  ≤30  31–45  46–60  61–75 | 83 (23.0)  128 (35.5)  107 (29.6)  43 (11.9) |
| Age, years, mean ± SD | 42.54 ± 13.80 |
| Smoking status, n (%)  Non-smokers  Ex-smokers  Current smokers | 226 (62.6)  86 (23.8)  49 (13.6) |
| BMI, kg/m^2^, mean ± SD | 26.07 ± 4.42 |
| BMI, kg/m^2^, n (%)  <25  25–29.9  ≥30 | 142 (39.3)  155 (42.9)  64 (17.7) |
| Occupational exposure to dust/fumes, n (%)  No  Yes | 277 (76.7)  84 (23.3) |
| Raise on a farm, n (%)  No  Yes | 338 (93.6)  23 (6.4) |
| Urbanization degree, n (%)  >10 000 inhabitants  ≤10 000 inhabitants | 270 (74.8)  91 (25.2) |
| Highest education level, n (%)  Less than high school  High school  Tertiary | 42 (11.6)  143 (39.6)  176 (48.8) |
| Family history of allergy or asthma, n (%)  No  Yes | 131 (36.3)  230 (63.7) |
| Current cat ownership, n (%)  No  Yes | 295 (81.7)  66 (18.3) |
| Cat ownership during childhood, n (%)  No  Yes | 251 (69.5)  110 (30.5) |
| Presence of current asthma, n (%)  No  Yes | 124 (34.3)  237 (65.7) |
| Presence of current allergic rhinitis, n (%)  No  Yes | 84 (23.3)  277 (76.7) |

^†^The given percentages were computed within each group (according to columns).

^‡^BMI= Body Mass Index, SD= Standard Deviation.

**Table S3.** The comparison of demographical and clinical characteristics of subjects monosensitized to secretoglobin and those only sensitized to lipocalins ^†^

| **Demographical and clinical characteristics, n (%)** | Monosensitization to secretoglobin^1^  *n* = 164 | Sensitization to only lipocalins^2^  *n* = 14 | *P*-value |
| --- | --- | --- | --- |
| Sex  Males  Females | 87 (92.6)  77 (91.7) | 7 (7.4)  7 (8.3) | 0.826 |
| Age, years  ≤30  31-45  46-60  61-75 | 44 (93.6)  54 (90.0)  42 (91.3)  24 (96.0) | 3 (6.4)  6 (10.0)  4 (8.7)  1 (4.0) | 0.782 |
| Smoking status  Non-smokers  Ex-smokers  Current smokers | 106 (93.0)  36 (92.3)  22 (88.0) | 8 (7.0)  3 (7.7)  3 (12.0) | 0.703 |
| BMI, kg/m^2^  <25  25-29.9  ≥30 | 65 (90.3)  71 (94.7)  28 (90.3) | 7 (9.7)  4 (5.3)  3 (9.7) | 0.564 |
| Occupational exposure to dust/fumes  No  Yes | 129 (92.1)  35 (92.1) | 11 (7.9)  3 (7.9) | 1.000 |
| Raise on a farm  No  Yes | 154 (91.7)  10 (100.0) | 14 (8.3)  0 (0.0) | 1.000 |
| Urbanization degree  >10 000 inhabitants  ≤10 000 inhabitants | 136 (93.2)  28 (87.5) | 10 (6.8)  4 (12.5) | 0.283 |
| Highest education level  Less than high school  High school  Tertiary | 21 (87.5)  56 (90.3)  87 (94.6) | 3 (12.5)  6 (9.7)  5 (5.4) | 0.418 |
| Family history of allergy or asthma  No  Yes | 49 (84.5)  115 (95.8) | 9 (15.5)  5 (4.2) | 0.015 |
| Current cat ownership  No  Yes | 150 (95.5)  14 (66.7) | 7 (4.5)  7 (33.3) | <0.001 |
| Cat ownership during childhood  No  Yes | 129 (92.8)  35 (89.7) | 10 (7.2)  4 (10.3) | 0.511 |
| Presence of current asthma  No  Yes | 75 (96.2)  89 (89.0) | 3 (3.8)  11 (11.0) | 0.079 |
| Presence of current allergic rhinitis  No  Yes | 38 (90.5)  126 (92.6) | 4 (9.5)  10 (7.4) | 0.743 |

^1^Participants with sensitization to only secretoglobin (Fel d 1)

^2^Participants with sensitization to only lipocalins (Fel d 4 or Fel d 7)

^†^The given percentages were computed within each independent variable (according to rows).

^‡^BMI= Body Mass Index, SD= Standard Deviation.
